# The TREAT-NMD advisory committee for therapeutics (TACT): an innovative de-risking model to foster orphan drug development

**DOI:** 10.1186/s13023-015-0258-1

**Published:** 2015-04-23

**Authors:** Emma Heslop, Cristina Csimma, Volker Straub, John McCall, Kanneboyina Nagaraju, Kathryn R Wagner, Didier Caizergues, Rudolf Korinthenberg, Kevin M Flanigan, Petra Kaufmann, Elizabeth McNeil, Jerry Mendell, Sharon Hesterlee, Dominic J Wells, Kate Bushby

**Affiliations:** Newcastle University, Newcastle upon Tyne, UK; Cydan Developments Inc, Cambridge, USA; PharMac LLL, Boca Grande, USA; Children’s National Medical Center, Washington, USA; Johns Hopkins University, Baltimore, USA; Genethon, Evry, France; University of Freiburg, Freiburg, Germany; Nationwide Children’s Hospital, Columbus, USA; National Institutes of Health, Washington, USA; Parent Project Muscular Dystrophy, New Jersey, USA; Royal Veterinary College, London, UK

**Keywords:** Neuromuscular disease, Rare disease, Review, De-risking, Drug development

## Abstract

Despite multiple publications on potential therapies for neuromuscular diseases (NMD) in cell and animal models only a handful reach clinical trials. The ability to prioritise drug development according to objective criteria is particularly critical in rare diseases with large unmet needs and a limited numbers of patients who can be enrolled into clinical trials. TREAT-NMD Advisory Committee for Therapeutics (TACT) was established to provide independent and objective guidance on the preclinical and development pathway of potential therapies (whether novel or repurposed) for NMD.

We present our experience in the establishment and operation of the TACT. TACT provides a unique resource of recognized experts from multiple disciplines. The goal of each TACT review is to help the sponsor to position the candidate compound along a realistic and well-informed plan to clinical trials, and eventual registration. The reviews and subsequent recommendations are focused on generating meaningful and rigorous data that can enable clear go/no-go decisions and facilitate longer term funding or partnering opportunities. The review process thereby acts to comment on viability, de-risking the process of proceeding on a development programme.

To date TACT has held 10 review meeting and reviewed 29 program applications in several rare neuromuscular diseases: Of the 29 programs reviewed, 19 were from industry and 10 were from academia; 15 were for novel compounds and 14 were for repurposed drugs; 16 were small molecules and 13 were biologics; 14 were preclinical stage applications and 15 were clinical stage applications. 3 had received Orphan drug designation from European Medicines Agency and 3 from Food and Drug Administration. A number of recurrent themes emerged over the course of the reviews and we found that applicants frequently require advice and education on issues concerned with preclinical standard operating procedures, interactions with regulatory agencies, formulation, repurposing, clinical trial design, manufacturing and ethics.

Over the 5 years since its establishment TACT has amassed a body of experience that can be extrapolated to other groups of rare diseases to improve the community’s chances of successfully bringing new rare disease drugs to registration and ultimately to market.

## TREAT-NMD and trial readiness in neuromuscular diseases

Neuromuscular diseases (NMD) are rare disabling conditions with an unmet medical need. Within the EU Framework Programme 6, funding was granted to a network of excellence “Translational Research Europe: assessment and treatment of rare inherited neuromuscular diseases” (TREAT-NMD), tasked with ‘reshaping the translational research environment’ for these conditions. As part of this effort, the TREAT-NMD partners developed infrastructures to support trial readiness for NMD, considering the unique challenges of therapy development for rare diseases. TREAT-NMD tools and resources included standardised operating procedures for the assessment of animal models [1,2], biobanks [3], patient registries [4,5], a care and trial site registry [6], generation and dissemination of care guidelines [7,8], evaluation and elaboration of outcome measures [9-11], regulatory interactions [12], and an active Project Ethics Council [13].

While the scope of TREAT-NMD includes all rare inherited neuromuscular diseases, its initial focus was Duchenne Muscular Dystrophy and Spinal Muscular Atrophy. In discussion with stakeholders a major gap in the field was identified. Despite hundreds of publications on potential therapies for these diseases based on work in cell and animal models [14], only a handful were moving forward into clinical trials, and there was no systematic way for such potential therapies to be evaluated for their development potential. In addition, where there was interest in progressing along a clinical pathway, expertise to do this was limited. Academic groups and small companies frequently lacked insight into the full implications of the drug development process, and even large pharma often lacked knowledge of the specific diseases, many with no development or regulatory precedent. Experience from other fields [15] suggested room for improvement in enabling therapeutic development in these rare conditions. Finally*,* while patient organisations and other funders were being asked to fund therapeutic development programmes, not all of them have established objective advisory system to appraise the clinical potential for candidates, independent from their existing internal scientific advisory boards who were responsible for making funding decisions.

### Establishment of the TREAT-NMD advisory group for therapeutics (TACT)

With these challenges in mind, TACT was established. A founding chair with experience of drug development and knowledge of the neuromuscular field worked with a secretariat based in the TREAT-NMD co-ordination office. Together they developed the composition and terms of reference of the group and launched TACT in 2009.

In the selection of the members of TACT, two major considerations were applied. The first was the requirement for multidisciplinary membership addressing the complex and multistep nature of the drug development process and the diverse areas of expertise it employs. The second criterion was excellence. In order for the committee to have credibility and to be used by the target community, the members recruited had to represent the global key opinion leaders in the field with genuine relevant experience. A “core group” representative of the different disciplines on the committee was established as a driver to oversee the work of TACT and guide its structure and content. All members of TACT signed a common confidential disclosure agreement, binding for all of the information received from applicants; conflict of interest statements are updated on a meeting-by-meeting basis.

### TACT membership and procedures

**Figure 1:**
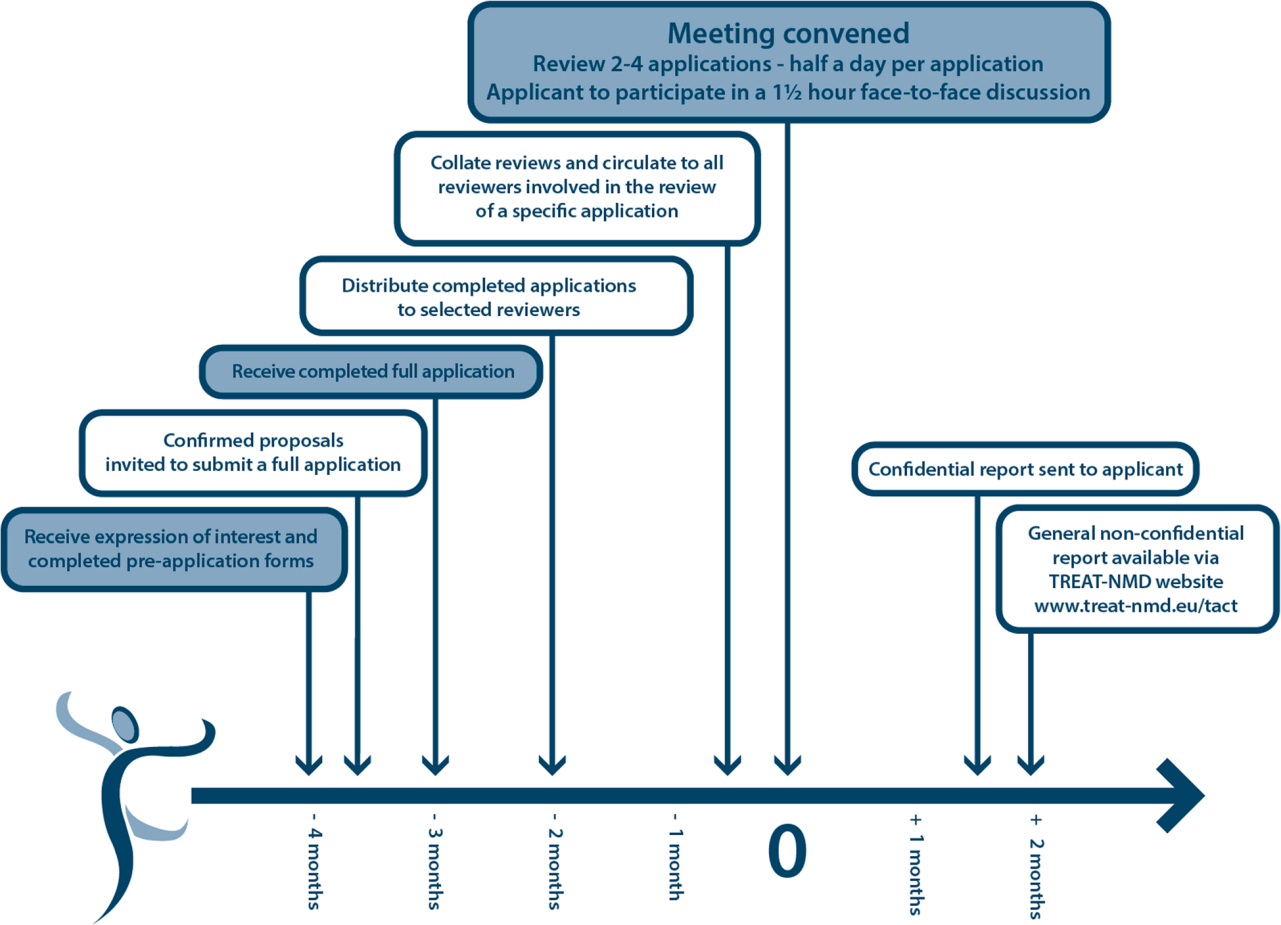
TACT meeting timeline.

Current membership of TACT includes 67 international experts in various areas of drug development, representing 11 countries. 29 members are from Europe (43.25%), 34 from the USA (50.75%), 3 from Australia (4.5%), and 1 (1.5%) from South America. Representatives from >10 patient groups have actively been involved with TACT since 2009, helping with the selection of projects, and providing part funding for costs. The committee meets twice yearly, with applicants in attendance. The discussion of each program takes half a day, and is preceded by up to 10 independent reviews by TACT committee members selected based on the most relevant expertise. Timelines are in place for the process of application, review and report generation (see Figure 1). A detailed application form was devised based on the process of drug development and designed to facilitate comprehensive appraisal (http://www.treat-nmd.eu/resources/tact/process/). Interested groups complete a pre-application form and based on the required expertise and a preliminary review, the TACT core group selects between two and four pre-applications - which are within the remit of TACT and at an appropriate stage of development -to submit a full application. Completing the application focuses the applicant on a number of important considerations critical to advancing a compound into the clinic and beyond.

Feedback to the applicants, integrated by a lead reviewer from all reviews, is in a detailed written report reflecting all aspects presented in the application and specific questions where the applicant seeks guidance. This report and specific recommendations are confidential, but applicants are encouraged to make the TACT review available to interested parties such as funders on request. A short non-confidential summary of the recommendations agreed with the applicant is posted on the TREAT-NMD website.

## Discussion

### Utilisation and outputs of TACT 2010–2014

To date TACT has held 10 review meetings, 6 in Europe and 4 in the US and reviewed 29 program applications from both academic investigators and industry in several diseases: Duchenne Muscular Dystrophy, Spinal Muscular Atrophy, Becker Muscular Dystrophy, Congenital Muscular Dystrophy, Inclusion Body Myositis and X-Linked Myotubular Myopathy.

Of the 29 programs reviewed, (Table 1) 19 (66%) were from industry and 10 (34%) were from academia; 15 (52%) were for novel compounds and 14 (48%) were for repurposed drugs; 15 (58%) were small molecules and 11 (42%) were biologics; 14 (48%) were preclinical stage applications and 15 (52%) were clinical stage applications. 3 had received Orphan drug designation from EMA and 3 from FDA (1 compound receiving both EMA and FDA designation).

**Table 1:** **Previous applications (Feb 2010 – October 2014)**

**Name**	**Country**	**Disease**	**Drug**	**Stage of Development**	**Industry/Academia**
Christopher F. Spurney, MD Children’s National Medical Center	USA	DMD	Losartan/lisinopril	Clinical	Academia
Emilio Clementi MD, PhD & Grazia D’Angelo, MD, PhD H Sacco University Hospital	Italy	DMD	Isosorbide dinitrate plus ibuprofen	Clinical	Academia
Giuseppe Vita, MD & Sonia Messina MD, PhD University of Messina	Italy	DMD	Flavocoxid	Clinical	Academia
Ana Ferreiro, MD Institute of Myology	France	CMD	N-Acetylcysteine	Clinical	Academia
James Symons, M.S., PhD Phrixus Pharmaceuticals Inc	USA	DMD	P-188	Clinical	Industry
Bradley L Hodges, PhD Prothelia Inc	USA	DMD	Laminin-111	Preclinical	Industry
Paul Higgins, PhD Paratek Pharmaceuticals	USA	SMA	Screen of a tetracycline library one to be selected as a final candidate	Preclinical	Industry
Fredrick Sachs, PhD Rose Pharmaceuticals	USA	DMD	GsMTx4	Preclinical	Industry
Mark Blaustein Halo Therapeutics	USA	DMD	Halofuginone (HT-100)	Clinical	Industry
Fabrizio Dolfi, MD, PhD NicOx SA	France	BMD	Naproxcinod (HCT 3012)	Clinical	Industry
Chris N Airriess, PhD California Stem Cell	USA	SMA	Human Embryonic Stem Cell Derived Motor Neuron Progenitors	Clinical	Industry
Fred Marin, PhD GMP-Orphan SAS	France	SMA	Satisma (sodium phenylbutyrate)	Preclinical	Industry
Urs Ruegg, PhD University of Geneva	Switzerland	DMD	Tamoxifen	Preclinical	Academia
Joel Braunstein, MD, FACC, MBA Tivorsan	USA	DMD	Biglycan	Preclinical	Industry
Paolo Bettica, MD, PhD Italfarmaco	Italy	DMD	Givinostat	Clinical	Industry
Dariusz C Gorecki MD, PhD University of Portsmouth	UK	DMD	P2X7	Preclinical	Academia
Joanne Donovan, MD, PhD Catabasis Pharma, Inc.	USA	DMD	CAT-1004	Clinical	Industry
Erica Reeves, PhD ReveraGen BioPharma, Inc.	USA	DMD	VBP15	Preclinical	Industry
Patricio Sepulveda Myostin Therapeutics Pty Ltd	Australia	DMD	Myostin	Preclinical	Industry
Peter Flynn, PhD Fate Therapeutics Inc.	USA	DMD	Wnt7a	Preclinical	Industry
Jon Tisley, PhD Summit PLC	UK	DMD	SMTC 1100	Clinical	Industry
Jens Schmidt, PhD University Medical Centre, Göttingen	Germany	IBM	DMF/BG12 & 1400 W	Preclinical	Academia
Denis Guttridge, PhD Ohio State University	USA	DMD	NBD Therapy	Preclinical	Academia
Carl Morris, PhD Pfizer Inc.	USA	DMD	Anti-GDF-8 Ab	Clinical	Industry
Mimoun Azzouz, PhD University of Sheffield	UK	SMA	Viral vector to deliver the SMN transgene	Preclinical	Academia
Richard Franklin, PhD Tarix Orphan LLC	USA	DMD	TXA127	Preclinical	Industry
Seth Porter, PhD FibroGen	USA	DMD	FG-3019	Clinical	Industry
Ennio Ongini, PhD & Gloria Vigliani, MD TrophyNOD	France	DMD	Naproxcinod	Clinical	Industry
Suyash Prasad, MD Audentes Therapeutics	USA	XLMTM	AAV8	Clinical	Industry

For full details see http://www.treat-nmd.eu/resources/tact/reviews/past/).

Applications have been received from 6 countries, 16 (55%) from USA, 12 (41%) from Europe and 1 (4%) from Australia.

### Learning points

Over the course of these reviews, a number of recurrent themes emerged frequently requiring advice and education including:

1. Preclinical: The need to use gold standard operating procedures for animal model experiments, for example, utilising SOPs available via TREAT-NMD for models of three neuromuscular diseases: (www.treat-nmd.eu/sopdmd) (www.treat-nmd.eu/sopsma) and (www.treat-nmd.eu/sopcmd). In a number of cases TACT also encouraged the applicants to ensure their results were reproducible through independent validation [16].
2. Regulatory: The need to seek orphan drug designation early in the process and arrange Pre-IND meetings (FDA) or Scientific Advice/Briefing meeting (EMA).
3. Formulation: The lead molecule, which an applicant is intending to take into clinical development, has to be the one which has been evaluated pre-clinically and for which supporting pre-clinical data are supplied. This consideration, if not addressed at an early stage, can add years to, or halt a development programme.
4. Repurposing: Challenges of repurposing were frequently highlighted – for example efficacy results from other population groups may not be relevant in the NMD population, reformulating for paediatric populations may not be straight forward and toxicity studies may not be sufficient for the NMD target population.
5. Clinical Trial design: Frequent examples of suboptimal trial design were seen - for example plans to use small populations to get results ‘quickly’ when power calculations suggested the need for a larger sample size, selection of inappropriate outcome measures and lack of awareness regarding specific IRB or other regulatory restrictions when selecting a paediatric population.
6. Manufacturing: Appropriate consideration to the scale of the drug development process (manufacture, upscale, partnerships for funding).
7. Ethics: Failure to consider the impact of the proposed trial design on the target patient population. Issues of moving forward with trials when the proposals were not likely to yield meaningful results. Problems of being both too carefree and too careful about the use of relatively invasive outcomes such as muscle biopsies.

### Summary

Feedback shows that TACT recommendations have helped investigators, including industry, in evaluating their potential compounds in a time sensitive and cost effective manner. Applicants are helped to consider the development program in a methodical fashion in order to inform clear go / no-go decisions and enable optimal use of funding and resources. Such thought processes are frequently new in an orphan drug environment. The process and application form have been mirrored by funding organisations such as patient groups in order to assist them in determining priorities for funding, with several applications being championed by patient organisations who see the TACT process as being complementary to their internal evaluation processes. The success of TACT cannot be measured by the number of successful compounds entering clinic, but rather by the systematic approach to the realistic prospects for a particular compound which, positive or negative can help the field and applicant.

The increasingly large number of industry applicants suggests that TACT may have a valued role in the field, including to pharma. Key to TACT’s added value is also that its review is not conducted with the goal of making a funding decision or as a group funded by the prospective sponsor, but rather as an independent, centralized review service on drug development potential that can then be made accessible to many different advocacy groups and other funding organizations. This focus on de-risking the process of proceeding to trial and or ultimate registration is distinct from the role of individual scientific funding boards.

The number of trials currently (January 2015) listed in www.clinicaltrials.gov for DMD (N = 143) and SMA (N = 116) indicates that the field has moved on during the 5 years since the establishment of TACT, though progress in drug approval remains disappointing. In addition to these diseases the committee is well equipped to address other neuromuscular diseases with large unmet need, where research is now identifying potential therapeutic opportunities, such as Charcot-Marie-Tooth (CMT). Other, often ‘rarer’, rare diseases are at a similar point to where the neuromuscular field was in 2009. Mechanisms are not clear as to how these disease communities might be assisted to move to a point where the International Rare Disease Research Consortium (IRDiRC) aim of 200 new therapies for rare diseases by 2020 [17] can come to fruition. As non-profit disease funding organizations are increasingly focused on highly translational drug development programs, the need for access to a very sophisticated diligence process beyond the abilities of a typical academic advisory committee has become more apparent. Although many organizations have been able to develop this expertise through strategic hires, enhanced committees and paid consultants, other organizations, particularly smaller ones, do not have the resources to perform this level of diligence. Also, building the same capability multiple times over to review the same projects is not efficient. The TACT model developed by TREAT-NMD helps meet this growing need for sophisticated drug development diligence by providing a central resource that is not “owned” by any single funding group, but is accessible to all. We believe that the global rare disease community, in addressing the ambitious aims set out by IRDiRC, should consider the benefits of a TACT like model to add structure to help to de-risk the process and educate the field. In rare diseases, the answer to effective therapies is not so much “more shots on goal” but “better shots on goal”—we simply can’t afford the standard high failure rate of clinical development programs. Patients are the most valuable resource in the process of RD drug development: we believe that the model developed by TACT is a way to improve the community’s chances of successfully bringing new RD drugs to registration and ultimately to market.

## Abbreviations

AFM: Association Française contre les Myopathies
CMT: Charcot-Marie-Tooth
CNMC: Children’s National Medical Centre
DPP: Duchenne Parent Project Netherlands
EAHC: Washington and an Executive Agency for Health and Consumers
EMA: European Medicines Agency
FDA: Food and Drug Administration
IRDiRC: International Rare Disease Research Consortium
NMD: Neuromuscular diseases
PPMD: Parent Project Muscular Dystrophy
SOPs: Standard Operating Procedures
TACT: TREAT-NMD Advisory Committee for Therapeutics
TREAT-NMD: Translational Research Europe: assessment and treatment of rare inherited neuromuscular diseases
